# Dr. Bradley, on Strangulated Hernia

**Published:** 1810-08

**Authors:** 


### *To the Editors of the Medical and Physical Journal.*

GENTLEMEN,

SINCE my last communication to you, on the subject of reducing Strangulated Hernia by the Taxis, I have met with five more cases, where gentle and long continued pressure has been practiced. In two of these, I succeeded, but in the rest I failed. These cases, though possessing neither novelty nor interest, much different from those formerly sent you, I have given you in detail. One of them, where the taxis failed, was so uncommonly circumstanced, as to entitle it more particularly to a place in your widely circulated Journal.

Hannah North, a married woman, aged 33 years, but who never had children, applied to me in the year 1806 for a swelling in her right groin, which she first discovered fourteen years before, and had in this interval applied to many surgeons, but without obtaining relief, or any satisfaction, as to its nature or probable consequences. One surgeon, a man of eminence, did actually salivate her for it, and declared her case venereal. Several others, from their manner of treating her, seemed to have similar suspicions. On the other hand, there were some who suspected it to be hernia, but acknowledged there was something mysterious in the case. From anxiety arising from this state of uncertainty, as well as by the imputation cast on her character as a modest woman, (for she then was single) from the supposition of her case being venereal, her life had become extremely unhappy.

On examination of the swelling, I could perceive two distinct tumours bordering on each other, and nearly in the same horizontal plane. The one situated exteriorly was hard, more circumscribed and prominent; the other interiorly riorly was softer, more diffused, and had a larger basis. This latter tumour frequently lessened when in a recumbent posture, but never entirely disappeared. She also made frequent attempts to reduce it by the taxis, but does not recollect she ever succeeded.

Being satisfied that one of these swellings, at least, was a hernia, notwithstanding the intricacy of the case, I recommended her to apply a steel-truss to this tumour *only*, but I believe this advice she neglected, for on the evening of the 7th of February, 1807, she was seized with symptoms of strangulated hernia. I saw her the following morning, when she complained of sickness and frequent vomiting, constipation of the bowels, and pain in the abdomen, which shot upwards towards the stomach, and downwards towards the vagina. Her belly was neither swelled, nor very painful to the touch; her pulse was 92 and soft, and her tongue a little, though slightly furred. She said the interior swelling in the groin was somewhat painful, had been down some days past, and was now at its largest size.

I made gentle pressure on this tumour for three hours, having previously ordered her five drops of the *tinct. opii* to be given every eight minutes. This remedy, and the manner of giving it in such cases, had been recommended to me by a friend. The aq. litharg. acet. c. was applied in the form of stupes, and pressure the rest of the day and the following night was continued at intervals. By midnight, from mistaking the directions that were given her, she had consumed half an ounce of her drops, but after the first hour of taking them, her vomiting and other distressing symptoms abated.

On the morning of the 9th, the vomiting and pain, attended with hiccough, returned with increased violence. Fifteen grains of the vitriolated magnesia were given her in a dissolved state; but this seemed to increase her sickness. The opium was again had recourse to in the same manner, and with nearly similar effects; and two drachms and a half of it were taken, before it was ordered to be discontinued, and pressure was again applied as before.

The pulse, during all this time, continued to increase in velocity, without any diminution of force; her thirst had likewise increased, and her tongue had become rather more furred; yet, notwithstanding, there did not appear any symptoms of considerable local inflammation. Her belly was neither tense nor much painful, and the tumour bore handling well.

On

On the morning of the 10th, she was evidently worse. Her pulse was at 120, but not weak, and her vomiting was frequent and distressing. Her thirst was also considerable, and her belly rather tense and somewhat painful; but still I could not perceive so much of that languor and deprivation of vital energy, which too often characterize and attend this affection. The tobacco-clyster was now administered, (a drachm and a half of tobacco being boiled in a pint of water ten minutes.) This caused considerable sickness, during which, pressure was again made on the tumour for half an hour, but without the desired effect. After the influence of the clyster had subsided, the operation was performed. The incision was begun a little above the upper verge, and carried down below the inferior margin of the tumour. The fascia was next carefully divided; after this, I was surprised on the hernial sac coming in view, to discover a substance attached to the inferior and exterior portion of the sac, thus 8. This primâ facie appeared to be glandular, and adhered to the sac so firmly, as to render it impossible to ascertain the point at which they were united. This appendage was of an eliptical shape, resembling a moderate sized walnut, both in form and magnitude, and was somewhat flatted on its anterior and posterior parts. An incision was made better than half through it transversely, which discovered its texture, and some blood-vessels apparently enlarged, passing into it, convinced me it was one of the inguinal glands in a diseased state.

Having previously exposed the bottom of the sac by dissection, I desired an assistant to draw it a little exteriorly, and I gently passed the fore-finger of my left hand upwards between the sac and aponeurosis. The adhesions, which were only partial, and extended upwards about three-quarters of an inch, gave way by very little force. Having passed the tip of my finger within *Gimbernat's* ligament, I with the bubonocele-knife divided it upwards, and a little inclining towards the pubes. The incision, as nearly as I could ascertain, was about one-third of an inch in length; and the act of making it, gave me a sense of feeling, as if the knife were dividing something ligamentous. This being accomplished, in a few seconds the sac became less and flaccid, borborigmi ensued, and the patient expressed herself greatly relieved. In order, however, to be certain that the hernia had entered the abdomen, I pushed up the sac as far through the aperture as possible, without violence, but found the gland, as well as the adhesions of the the sac to the contiguous parts, considerably to impede my attempts. On handling the sac, I thought it much thickened; and I now made some attempts to separate it from the gland with my fingers, but found this also impracticable.

Being now satisfied the object of the operation was attained, the integuments were brought together over the gland and sac, by three stitches of the interrupted suture, assisted by straps of adhesive plaister, so as completely to unite the wound by the first intention. Nothing further occurred relating to the cure, of any importance. The wound healed kindly, and was attended with very little pain; and in twelve days was completely cicatrized.

The pulse, at the time of the operation, was at 122; an hour after, at 108; and the day following, at 96. In short, she rapidly regained her health.

It may be a matter of surprise, that the gland was not removed at the time of the operation, on account of its diseased state; but as it had preserved its texture, and exhibited no marks of disease, save enlargement; and as extirpation could not have been attempted, without probably leaving a part of it behind, or otherwise wounding the sac, I therefore suffered it to remain, being persuaded the operation would effect some change towards its annihilation or dispersion. This idea was eventually realised; for on the 15th of September, 1808, I was called to this patient, who complained of great pain in the hernial sac, but which was empty. Her pulse was at 103; she was thirsty; and her tongue a little furred. She was also excessively sick, but had not vomited, yet had retched a few times. Half a drachm of the vitriolated magnesia, dissolved in a table-spoonful of cold water, was ordered her every hour. In ten minutes after taking the first dose, her pain and sickness ceased; and after repeating the medicine a few times, it operated, and she ailed no more. On examining the groin, I could perceive no remains of the gland. She informed me, it vanished in a few weeks after the operation; and that the hernia now descended and receded, accordingly as it was favoured by the position of the body, more readily than formerly. From which time, to the present, she has worn a steel-truss, and continued in good health.

This case affords no *data* for determining the effects of pressure, for it is plain, from the frequent and ineffectual attempts of the patient formerly, at different times, to return the hernia, and the circumstance of the sac adhering to the inguinal gland, which prevented it being pushed up towards the stricture, that no pressure, on the principle of pressure, formerly recommended, could have much effect.

I have long been impressed with an idea, that under some circumstances, the hernial contents might with propriety be returned into the abdomen, without a division of the sac, being sensible of the advantages that would accrue from, and the inconveniences that would be obviated by, such a practice. Under this conviction, I have ventured to deviate from general custom; and although I have great, and almost general authority against me, yet the few observations I shall adduce on this practice, will, I trust, not only apologize for my conduct, but may call forth the more experienced part of the profession to a further discussion of this subject.

Two ostensible advantages apparently result from a division of the hernial sac. The first is, the greater certainty of removing the stricture; the second is the opportunity afforded for inspecting the hernial contents. As to the division of the sac, for the first intention, I believe it may frequently be obviated by the stricture being otherwise removed; and as the method of dividing the sac is counterbalanced by such important disadvantages, which are nothing less than rescuing the patient from one danger and exposing him to a second, any method that will effect the one object and prevent the other, is a desideratum in Surgery of no small moment.

What has confirmed modern surgeons in their idea of the necessity of uniformly dividing the sac, seems to have arisen from a contrary practice, adopted many years ago by some eminent surgeons, but which grew into disrepute, principally by their not attending strictly to the circumstance of ascertaining whether the stricture was overcome. They imagined, when the tendon of the oblique muscle was divided, that the stricture was of course removed. They found, however, on examining into the cause of the miscarriage, which in some instances happened, that the neck of the hernial sac in general formed the stricture, and was the source of the mischief. Hence, the old method of dividing the sac was reverted to promiscuously, without adopting or retaining any modification of the new practice; the utility of which, had, in many instances, been conspicuously evident.

By the non-division of the sac, the patient is certainly exposed to some risk from the neck of the sac forming the stricture, as has sometimes been the case; and it is pretty certain, certain, that in a great majority of the patients, who have died after the operation, where the sac has not been opened, it has *been* from this cause; to ascertain and remedy which, I believe in most cases, will not be difficult. After dividing the integuments, and properly exposing the sac, I would desire an assistant gently to pinch up the sac with his finger and thumb, in order to prevent it slipping into the abdomen; a circumstance that might possibly happen, but as the sac most generally adheres to the surrounding parts, this part of the operation will mostly be rendered unnecessary. Immediately after this, I would divide the supposed stricture; namely, the tendon of the oblique muscle in the inguinal, and Gimbernat's ligament in the femoral hernia. This being effected, the protruded parts, especially if the patient be laid in a posture favourable for reduction, will in most instances readily recede. The attainment of this object is generally known by a diminution of the pain and tumour, attended frequently with borborigmi, and succeeded by an alteration in the countenance, and particularly a sanguine manner in which the patient seldom fails to express his relief; but if, on the other hand, after waiting a while, any doubts of the stricture being removed still remain, it will be then necessary to open the sac. The few solitary instances we meet with in surgical authors, where some part of the hernial contents form the stricture, can have little weight towards pointing out the necessity of opening the sac, when set in opposition to the disadvantages resulting from that part of the operation.

Hence, if on dividing the tendon, every symptom of strangulation cease, together with a reduction of the hernia, and the sac at the same time remains unreduced, we may rest assured that the stricture is not in the neck of the sac. If the circumstance of the hernial contents forming the stricture, were not a very rare occurrence, a mode of operation, perhaps, preferable to the above, might be suggested; this scheme, though not founded on experience, is the result of some reflection. The method which I allude to for determining whether the stricture be in the protruded parts, is as follows. After the integuments in bubonocele are divided, and the sac properly denuded, the patient's body is to be elevated into a half erect posture, so that the axis of his trunk may form an angle of about forty-five degrees with the table on which he is laid. By this position, the hernia will be prevented from spontaneously ascending. The tendon is next to be divided, and in such a manner, if the cause of the mischief, as to leave no doubt of the stricture being removed. If all the symptoms of strangulation should now cease, it is conclusive, that the stricture is in the tendon of the oblique-muscle, and the hernia is to be returned into the abdomen, as before described. If, however, after waiting a while, any doubts of the stricture being removed are still entertained, a fair opportunity offers for opening the sac, for the purpose of discovering the stricture. The advantages which this method seems to possess, are sufficiently obvious. First, it ascertains whether the stricture is in the tendon, or in the sac, or some part of its contents; if in the former, then the hernia may be returned, without a division of the sac, where the object is to remove stricture only; if in the latter, then the real necessity for opening the sac is indicated.

In irreducible hernia, in an incarcerated state, by the non-division of the sac, we have fewer means in the operation of ascertaining the removal of the stricture, than when the parts are capable of being returned into the abdomen. The feelings of the patient, and a suspension of the symptoms, are our principal criteria. Notwithstanding these scanty means of discrimination, before we expose our patient to the consequences of dividing the peritoneum, we ought to wait a while, as before mentioned in reducible hernia, after the division of the integuments and tendon if the circumstances of the case will admit it, and if a total cessation of all the unpleasant symptoms should ensue, for, we will suppose, the space of an hour, we should not hesitate to unite the wound by the first intention.

This practice of waiting may appear trifling to the patient or bye-standers; but to the humane surgeon, who is fully impressed with the consequences dependent on his conduct, and who wishes to avoid incurring danger, rather than try the experiment of overcoming it, this practice, I hope, will have its due weight. As this method also can only be advisable, when our object is to remove stricture, and as this must often succeed, without exposing the patient to those consequences arising from a division of the sac, such an attempt, guided by caution and judgment, ought in my opinion to precede this most serious part of the operation.

The reasons for not opening the sac, in reducible, apply equally or with greater force to irreducible hernia. For in the latter, as soon as the stricture is removed, we are to consider the prolapsed parts, either in a state of inflammation tion or gangrene. If in the former, then the advantages will be greatly in favour of not opening the sac, for reasons that are obvious. If, on the other hand, mortification exist within the sac, it is in general confined to parts below the ring; and the enveloping covering will impart such warmth to the diseased hernia, as cannot fail not only to arrest the progress of mortification, but to expedite the process of separating the gangrenous from the sound parts. Hence, as this business cannot be effected without suppuration, a sanious matter will form, and accumulate in the sac, which, if not evacuated by an artificial opening, and left to nature, will be discharged by a spontaneous rupturing, attended with considerable loss of substance. On the contrary, if the practice of opening the sac be adopted, at the time of the operation, and the morbid part of its contents be removed by the knife, the consequences are frequently fatal; and, if even they are only in a state of inflammation, from their being previously thickened and diseased, any new stimulus applied, or additional violence done to them, must cause the subsequent inflammation to be alarmingly great.

The advantages necessarily resulting from this unusual mode of operation, as above recommended, are such as have perhaps not been duly appreciated. Air, when admitted into the abdomen, has been long considered as deleterious; and the division of the sac, in a state of irritation, which is the peritoneum itself, must in conjunction with this circumstance, be sufficient greatly to aggravate all the distressing symptoms which existed at the time of the operation. Whoever has seen the difference betwixt wounds in the abdominal covering, where the peritoneum has been divided, and otherwise, will be satisfied, this membrane cannot be injured without an increase of subsequent inflammation; but the most convincing of all arguments will be, the contrast between these cases, where the operation has been performed in the usual way, and those where the taxis has succeeded. This difference in their result is so striking, as may draw the attention of surgeons, at some future period, to a more able consideration of this subject. Mr. Wilmer gives us a number of apparently desperate cases, where the hernia was reduced without the assistance of the knife, and the patients ailed no more; and he also gives us other cases, where the operation of dividing the sac was performed in the usual way, and a great proportion of the patients died. Mr. Hey seems to have been no more successful in his practice; yet both these gentlemen, as well as as others, have attempted to reduce the hernia, previous to the use of the knife, therefore their object in dividing the sac, could not be for the purpose of inspecting the hernial contents, but for removing stricture only; and as this may generally be effected without a division of the sac, it follows, that I was justified in performing the operation, as above described.

From the preceding premises, as to the propriety for and against opening the sac, the following inferences may be deduced.

1st. That a great proportion of those cases, where the sac has been opened, and the hernial contents have been in a diseased state, have terminated unfavourably.

2dly. That a great proportion of those cases, where the stricture has been removed, without a division of the sac, have done well.

3dly. That in all those cases, where the taxis is performed, the surgeon is justified in returning the hernia by any other method, save that of opening the sac; because the taxis implies, that the parts are in a fit state for reduction.

4thly. It also follows, that if the method above described, of returning the hernia, without opening the sac, be unjustifiable, the operation of the taxis immediately preceding it, is unwarrantable.

5thly. That if the state of the case require the hernial parts to be inspected, any attempts at reduction, immediately preceding the operation, will be inadmissible.

6thly. That in most instances, where the operation is performed, without dividing the sac, and symptoms of stricture still remain, the seat of the mischief is generally in the neck of the hernial sac, which is to be opened accordingly.

As to the theory of returning the sac unopened, I wish to be understood in a qualified sense; and in order to prevent misconception, I will be as brief and explicit as possible.

In all cases, where the surgeon on due deliberation, judges the hernial contents to be in a fit state for reduction, and performs the taxis accordingly; in such cases I would not hesitate to adopt the practice, under the precaution and rules before described; and it is on this ground only, I wish to advise or recommend it. Again, if the taxis be right, and it fail, this method cannot be wrong; or, in other words, if it be right to perform the taxis, can it be wrong to return the hernia, with the sac unopened, by an operation? Where is the difference? In the the one instance, the stricture is overcome by the hand; in the other, it is removed by the knife. Are not the symptoms, peculiar to reduction, common to both methods? Are not these common symptoms as conclusive in one case as the other? What surgeon will hesitate to speak decidedly, when he succeeds by the taxis, as to the certainty of the hernia being reduced? What reasons, therefore, will he have to doubt the return of the hernia by the other method? Cannot he ascertain the reduction in one instance as well as the other.

( To be Continued. )

